# A Prospective Six-Month Study of Chronic Pain Sufferers: A Novel OTC Neuromodulation Therapy

**DOI:** 10.1155/2019/3154194

**Published:** 2019-09-30

**Authors:** Richard Staelin, Sree N. Koneru, Ian M. Rawe

**Affiliations:** ^1^Duke University, Durham, NC, USA; ^2^BioElectronics Corporation, Frederick, MD, USA

## Abstract

**Objective:**

To assess the durability of treatment over various chronic pain conditions of an emerging, nonprescription electromagnetic neuromodulation device that uses pulsed shortwave therapy.

**Methods:**

A 6-month prospective study, involving 240 chronic pain sufferers, 94% of whom reported using pain pills and 98% reported using pain therapies prior to entering the study. Their average baseline pain was 8.2 VAS points before treatment; they had a pain duration of 6.5 years, and they were positive responders to pulsed shortwave therapy in an initial 7-day trial. Prospective assessments were obtained at intervals of 3, 4, and 6 months following a retrospective 7-day assessment. Longitudinal analyses were conducted to determine pain relief trends after the initial 7-day device use.

**Results:**

Seven days after initial treatment, the average pain was reduced to 2.9, a 65% pain reduction for the study subjects. At the 6-month measurement, the average pain was 3.3, a 60% pain reduction from baseline. Only 17% of the subjects saw their pain level increase although this new level was still lower than baseline pain. Pain relief translated into improved quality of life and reduced medication use for the majority of the subjects. There were no significant adverse side effects reported over the 6 months of use.

**Conclusion:**

Ninety-seven percent of the recruited subjects, all of whom had previously reported clinically significant pain relief using the 7-day PSWT device, sustained this relief for 6 months by using the device on an as-needed basis.

## 1. Introduction

Developing long-term effective treatments for chronic pain sufferers has proved to be elusive. Evidence from clinical trials and systematic reviews indicate that many interventions for chronic pain provide only mild-to-moderate short-term benefits, with a lack of evidence for long-term effectiveness [1]. The challenge with treating chronic pain is reflected in the lack of correlation between pain level and severity of tissue damage [2] due to complex changes in immuno, sensory, hormonal, and inflammatory processes in the peripheral and central nervous system. Repetitive nociceptive stimulation induces pathophysiological changes in the pain pathways leading to a persistent state of high reactivity and a lowering of the pain threshold. Such a condition is referred to as central sensitization (CS) [3, 4]. Often this occurs after the onset of persistent acute pain which then transitions to chronic pain and is marked by CS-associated neuroplasticity. CS has been linked to the etiologies of osteoarthritis [2, 5], chronic lower back pain [6], plantar fasciitis [7], fibromyalgia [8, 9], neuropathy [10], migraine [11], and many other chronic conditions [6, 12, 13].

Recent guidelines on treating chronic pain recommend a multimodal treatment approach, with an emphasis on nonpharmacologic therapies prior to using pharmacological treatments [14]. Bioelectronic medicine is one such treatment approach aimed at providing therapeutic benefits and involves the use of electrical, magnetic, optical, and ultrasound pulses to modulate nervous system activity (*neuromodulation*) [15]. Those devices that use electrical impulses to achieve targeted neuromodulation are referred to as “*Electroceuticals*.” They do this through at least three methods: invasive (implanted), semi-invasive (surface electrodes, such as in transcutaneous electrical nerve stimulation (TENS) devices), or noninvasive (using electromagnetic fields (EMF)). Modalities based on the first two approaches, i.e., spinal cord stimulators (SCS) and TENS, are routinely employed for pain management [16]. However, these invasive and semi-invasive electroceuticals present risks such as skin damage, postsurgical complications, and cost, factors which have largely tempered recurring use of these electroceuticals for chronic pain management.

The third electroceutical approach relies on EMF. This has the unique advantage of not requiring direct skin contact and thus can be used over clothing/bandaged skin, etc. In addition, the use of radiofrequency (RF) EMF (MHz range) potentially allows battery-operated electroceuticals with long lifetimes. Although classic bioelectromagnetic theory indicates that beyond 10 MHz, RF fields are incapable of producing biological effects other than simple heating [17], Koneru et al. have demonstrated that when low-power RF transmitters are operated adjacent to biological tissue and at maximum output (saturation), modulation of peripheral nerve activity (neuromodulation) can occur [18]. This indicates that RF EMF electroceuticals can achieve neuromodulation.

Pulsed shortwave therapy (PSWT) is a low-power RF electroceutical technology that operates at saturation and relies on tissue absorption of EMF to achieve neuromodulation of peripheral nerves [19, 20]. Recently, a wearable version of PSWT, sold under the brand name ActiPatch®, has become available for nonprescription use in the United States for treating knee osteoarthritis and plantar fasciitis, both of which have been linked to CS. At the time of this study, it was available as a 7-day unit (with no on/off switch) or a 30-day unit with an on/off switch. Although this device does not provide any sensory feedback when topically placed over the area of pain, the PSWT device has been shown to reduce chronic pain and improve quality of life for several chronic pain conditions over treatment periods ranging from 7 days [21–23] to 28 days [24].

At least 3 studies have investigated treatment effectiveness of this device over 7 days. One is a randomized controlled trial (RCT) conducted in the US on plantar fasciitis [21], and two are large registry studies of UK pain sufferers, most of whom reported suffering from pain for at least 6 months prior to using the device. Each study assessed pain using a 0–10 point visual analog scale (VAS). In the plantar fasciitis study, which consisted of 70 subjects, the active treatment group reported a 40% reduction in morning pain (1.7 VAS points) following 7 days of PSWT use, compared to only 7% in the placebo group (0.3 VAS points). The first of the registry studies reported on 5000 subjects suffering with chronic pain of varying etiologies [22]. The study population consisted of individuals with severe pain levels (average VAS ≈ 8) who had independently purchased and used a trial unit (the 7 day unit) of the medical device. Sixty-five percent (65%) reported a clinically meaningful reduction in pain (defined herewithin to be ≥2 VAS points) [22]. In this subgroup of clinically significant responders, the mean reduction in pain was 57% (4.7 VAS points). The second registry study involved 1394 chronic back pain sufferers, who reported baseline measures that were very similar to the first registry study [23]. In this back pain study, 52% reported at least a 40% reduction in pain, resulting in an average pain reduction of 5.4 VAS points after 7 days of treatment [23].

A second RCT study, consisting of 60 subjects, investigated treatment efficacy over 28 days of daily PSWT device use, in reducing chronic knee osteoarthritis pain and changes in function. Subjects receiving active treatment reported a 25% decrease in VAS compared to only 3% in the placebo device group [24]. Additionally, active treatment subjects reported a 16% improvement in functionality when compared to 1.6% in the placebo group. More importantly, subjects in the active treatment group demonstrated a significantly greater improvement in pain tolerance thresholds, via a technique known as quantitative sensory testing (QST). This approach is widely considered to be a gold standard in quantifying nerve hyperactivity associated with CS [25].

The above-discussed literature indicates that daily PSWT use is effective in reducing chronic pain levels for a majority of pain sufferers with varying etiologies (many of which are associated with CS) and for up to 28 days. However, evaluating treatment durability for longer periods is crucial in determining the durability of treatment effectiveness. A decline in treatment effectiveness is commonly associated with long-term use of pharmacological treatments, for example, with NSAIDS [26] and opioids, owing to tolerance [27]. The goal of this prospective study is to assess the durability of treatment effectiveness for the PSWT medical device over a 6-month period. Specifically, we investigate whether subjects who reported clinically significant pain relief following 7-day use of the medical device were able to maintain longer-term relief over 6 months, and if so, were any factors predictive of the magnitude of pain relief. The study sample was composed of 240 chronic pain sufferers who had previously indicated that they had been suffering with pain for at least six months, had already started using the 7-day PSWT device, had lowered their pain level by a minimum of 2 VAS points over the course of this 7-day treatment, and had intended to continue treatment using the longer-lasting device. These subjects were then assessed over six months of treatment, evaluating changes in pain level, functionality (sleep and physical activity), quality of life, and medication use compared to baseline.

## 2. Materials and Methods

This study is a prospective study and was carried out over 6 months. Subjects provided consent, and data analysis was approved by the Institutional Review Board of Duke University (2019-0285).

### 2.1. Population and Study Sample Characteristics

The sample for this prospective study came from a population of 1841 UK/Ireland chronic pain sufferers who independently purchased a 7-day trial version of the focal PSWT medical device between April and October of 2015 and who also responded to a follow-up marketing and assessment e-mail sent out by the manufacturer. This initial assessment determined, among other measures, the individual's baseline pain level, the duration of this pain, the pain level after 7 days of PSWT, current treatment therapies, and the degree of their intention to continue treatment with the medical device. The average baseline pain for this population before using the medical device was 8.0 VAS points. They also reported being in pain for an average of 6.4 years and prior to using the medical device were using an average of 1.8 therapies that included pain pills (85%), TENS (16%), heat wraps (27%), topicals (33%), and physical therapy (20%). Of these users, 1143 (62%) reported clinically significant pain reduction over the course of 7 days of PSWT and of this clinically responsive subset, 682 (60%) indicated a definite intent to continue therapy by purchasing the retail (longer lasting) PSWT device. This latter subset was contacted via e-mail with a request to consent in participating in a 6-month study and complete three further assessments. No restrictions were placed in terms of them using or discontinuing other therapies during the study period or the degree to which they needed to use the medical device. The only requirement was that they were asked to fill out the three assessments, which were measured among other things, their use of other therapies, and the degree to which they used the medical device. Of the 682 subjects contacted, 240 (35%) agreed to participate in the study, provided written consent via e-mail, and completed at least one additional assessment. Subjects who completed the six-month assessment were compensated with a free 720-hour retail version of the device (retail price £19.95) given at the end of the study.

In summary, the prospective sample consisted of long-term pain sufferers who, prior to using the medical device, had not found (or at least were not using) therapies that reduced their high levels of pain, but who after using the device for 7 days, reported clinically significant short-term pain relief, who continued use after purchasing the commercially available 30-day device and who provided consent and participated in the prospective study.

**Figure 1:**
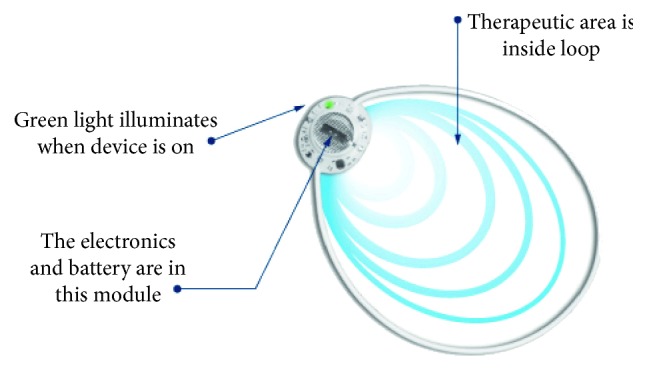
ActiPatch® is a commercially available, topically applied, over-the-counter medical device used for treating chronic pain. The device provides stimulation that relies on tissue energy absorption of high-frequency electromagnetic waves to influence nerve activity in the exposed tissue, a process known as neuromodulation (reproduced with the permission of BioElectronics Corp).

The PSWT medical device used in the study is commercially available (ActiPatch®, BioElectronics Corporation, Frederick, Maryland USA). It is regulated as a class II device (special controls) by the US FDA and indicated for over-the-counter use in treating chronic musculoskeletal pain related to knee osteoarthritis and plantar fasciitis in the US. It is also available for broader use in Canada, EU, Australia, and many other countries where it is regulated as a class II(a) device. The device operates at a carrier frequency of 27.12 MHz and has a pulse frequency of 1 kHz, each pulse sustained for a duration of 100 microseconds. The device has a peak incident power of 73 *μ*W/cm^2^ (as measured into a 50-ohm load) and a treatment area of 110 cm^2^ (See Figure 1).

### 2.2. Primary Outcome Measures

The goal of this study was to determine if initial pain relief, measured in terms of pain reduction over the 7-day treatment period, was maintained over a period of 6 months. This longer-term pain relief was measured not only by the six-month pain level and changes in pain level from baseline pain but also by changes over time in function (sleep quality and physical activity), quality of life (QoL), and medication use. Using multiple outcome measures reflects the belief that pain relief is a multidimensional construct. Consequently, all of these change measures were viewed to be primary measures of longer-term pain relief.

### 2.3. Description of Assessments

Subjects were sent an assessment at 3, 4, and 6 months following their initiation of PSWT. As a result, four data sets were potentially available for each of the 240 subjects that participated in the proscriptive study. In the first assessment, subjects' demographics were collected, as well as the location of device use (back, knee, etc.), the underlying etiology, the location of their pain, the baseline pain level, the pain level after 7 days of treatment, the use of analgesic medications, the use of alternate pain therapies (TENS, heat wraps, topicals, physical therapy, and other), and the intent to purchase the longer-lasting medical device. There were no data collected on the stage and/or classification of the chronic pain condition other than pain level, duration, and location of pain. In the three follow-up assessments, data were collected on current pain level, how often they used the medical device, and the degree of change (if any) from baseline in sleep quality, physical activity, quality of life, medication use, and other pain therapies. The levels of available responses and the coding for each response for many of the questions asked are given in Table 1. Higher numbers indicated a more positive change, i.e., an increase in sleep, physical activity, and quality of life and a decrease in medication use and the use of other therapies.

### 2.4. Data Preparation and Statistical Analyses

Raw data were collected using the Constant Contact e-mail application (*Constant Contact Inc., Waltham, Massachusetts USA*) and exported into a comma-delimited (CSV) file and analyzed with Excel 2016 (*Microsoft Corporation, Redmond, Washington USA*). Data from the 4 assessments times (day 7, 3 months, 4 months, and 6 months) were merged to yield one longitudinal database from which subject identification information (e-mail address) was removed. The Institutional Review Board of Duke University provided protocol approval under Analysis of Existing Data for this database along with the sample of 1841 trial device users.

Temporal changes in outcome measures were analyzed two ways: (1) changes in pain levels (VAS), which were determined by calculating the difference between a subject's baseline pain (day 0, prior to using the trial device) and their reported current pain; (2) Changes in the other measures (i.e., sleep quality, physical activity, medication use, and overall quality of life), which were determined by the subject's responses to questions concerning the extent of change over the specified assessment period, relative to the person's baseline (Table 1). The basic assumption in making these temporal comparisons is that although there is no comparison group not using the medical device for six months, each individual acts as his or her own control since subjects had been treating their pain beforehand, had been suffering from this pain for a long time (average 6.5 years), and their high baseline pain levels implied they had not obtained any substantial long-term temporal relief in pain level from the other tried therapies. We later control for therapy use and duration of prior pain when assessing changes in pain relief after using the medical device during the study period.

Any missing data during the six-month period for an individual were imputed using the last-observed carried forward (LOCF) approach. Factors associated with each of the different multidimensional change measures (including medication use) were determined via OLS regression analyses using Regressit, an Excel add-on statistical package. A *p* value of 0.05 was set as the threshold for determining statistical significance. Analyses were also conducted to see if there was any selection bias or bias due to subjects dropping out of the study. This was done by comparing the characteristics and distributions of pain levels across three different subsamples of the population of 1841 chronic pain sufferers, these groups being the retrospective population of 1841 users of the 7-day device, the sample of subjects who completed the six-month assessment, and the sample of subjects that dropped out of the study before completion.

## 3. Results

### 3.1. Description of Study Sample

The study sample had an average age of 57.9 years, were primarily women (70%), and predominantly (91%) had pain for more than 6 months at the beginning of the study (average 6.5 years). These demographics are nearly identical to the demographics for the sampled population (Table 2 comparing columns 1 and 6). The etiologies most commonly reported by subjects in both the study sample and the sampled population were arthritis and fibromyalgia. However, the study sample reported a higher incidence of fibromyalgia (20%), when compared to the sampled population (10%). While baseline pain levels (VAS) were similar in both groups, the VAS pain levels following 7-day treatment with the PSWT were markedly different (5.0 for the sampled population vs. 2.8 for the study sample). No major differences were observed between the two groups in terms of demographics except by gender (Table 2). This difference is due to the fact that the subsample indicating clinically significant reduction in 7-day pain contained 70% women, compared to only 61% for the subsample not reporting a minimum 2 VAS pain reduction after 7 days (see columns 2 and 3.) This latter subsample also reported lower baseline pain (7.77 vs. 8.17) and a much smaller intent to “definitely buy” a retail device (60% vs. 3%).

### 3.2. Prior Medication Use in Study Sample

Initial assessment of medication at baseline shows that 94% of subjects in the study sample were using pain pills and 98% were using some pain therapy. A subsample of size 172 of the study sample provided more detail on the variety of analgesic medications they used to help with their severe pain level (Table 3). Ninety-five percent (95%) reported using at least one OTC or prescription analgesic and 43% indicated using at least one opioid or morphine medication.

### 3.3. Missing Data

Of the 240 subjects, 31 did not complete all follow-up assessments. Of these 31, 15 subjects completed only the 3-month assessment, while the remaining 16 completed both the 3-month and 4-month but not the 6-month assessment. Subgroup analysis for these 31 subjects indicated that 15 last reported having mild pain (0–3 VAS) and their quality of life had improved “a great deal” or shown “a definite improvement.” In contrast, the remaining 16 last-reported VAS scores ≥4 before being lost to follow-up and many of these 16 subjects reported little or no improvement in their quality of life.

### 3.4. Temporal Trends in Pain Reduction

**Figure 2:**
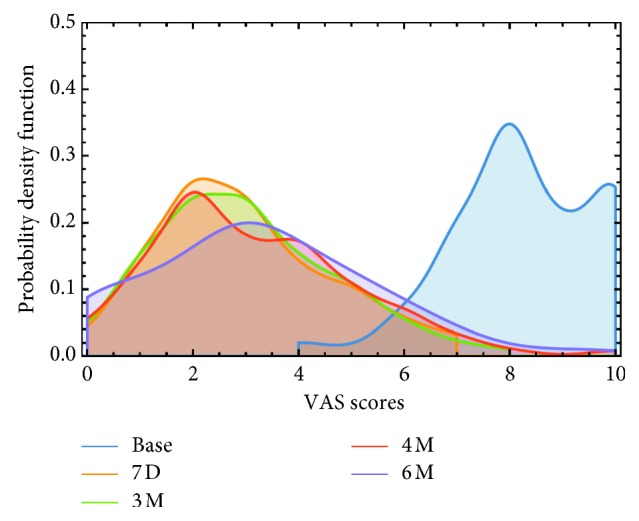
Distribution of pain levels (VAS) in the study sample at the various time points during which data were collected: baseline, 7days, and 3, 4, and 6 months. A shift in distribution from baseline, following initial 7-day PSWT treatment, indicates that most of the pain relief obtained in the first 7 days is maintained over a 6-month period, with continued device use.

The distribution of subjects, partitioned by three pain categories for the different points in time, is shown in Table 4, while the full distribution of pain scores for these same time periods is shown in Figure 2.

Ninety-one percent of the study sample reported being in severe pain prior to using the medical device (baseline), but only 3% continued to be in severe pain after the 7-day PSWT treatment period. In contrast, the subsample of the population who did not indicate clinically significant initial reduction reported almost no average pain reduction over this time period (see Table 2). By the end of the 6-month intervention period, using data for 209 subjects who completed the assessment in month 6, 58% reported being in mild pain, 36% reported moderate pain, and 6% reported severe pain. Importantly, 9% reported no pain. These lower levels of pain (and thus large pain reductions) are in stark contrast to the high pain levels these subjects reported having for extended periods of time (average duration of 6.5 years) prior to using the medical device.

Comparing the breakdown of scores for the 209 subjects with that of the 31 subjects who did not complete the study, we found 52% of the latter group reported mild pain on their last assessment, 42% moderate pain, and 6% severe pain. These percentages are very similar to the percentages in each of the pain categories for the 209 subjects that completed the six-month assessment. Consequently, the distributions for all 240 subjects used in the study are very similar to the 209 who completed the last assessment.

The vast majority (72%) of the subjects, who reported mild pain levels after 7 days of treatment, reported mild levels of pain at the end of the six-month study period, and the remaining 28% reported moderate pain levels (Table 5). For the other two 7-day pain levels (i.e., moderate and severe), the general trend for the duration of the study was towards lower pain levels (i.e., pain reduction). For example, all but 9% of those few who reported severe levels of pain after 7 days reported mild (36%) or moderate (55%) levels of pain by the end of the study.

### 3.5. Additional Outcome Measures

Subjects also provided other measures of pain relief, i.e., improvements in function (sleep quality and physical activity) and overall quality of life, any decrease in their medication use, and stopping medication use or other therapies (Table 6).

These additional outcome measures show strong associations with the initial 7-day pain level and the final pain level. The 57% of subjects in the study sample who reported being in the mild pain category by the end of the study also reported an average QoL score greater than 5 (“*=definite improvement, one that made a real difference*”) and average scores greater than 2 for sleep and physical activity (“*=improving a fair amount*”). Approximately 28% of the study sample indicated that they were no longer using any analgesic medications and 16% stopped using other therapies; unsurprisingly, subjects with the lowest final pain levels more likely belonged to these groups. Even the 15% of subjects in the study sample who reported final VAS≥6 reported functional improvements: ≥3.5 for QoL (“*=a slight but noticeable difference*”) and ≥1.00 for sleep quality (“*=little improvement*”) although all these individuals continued to use pain medication.

**Figure 3:**
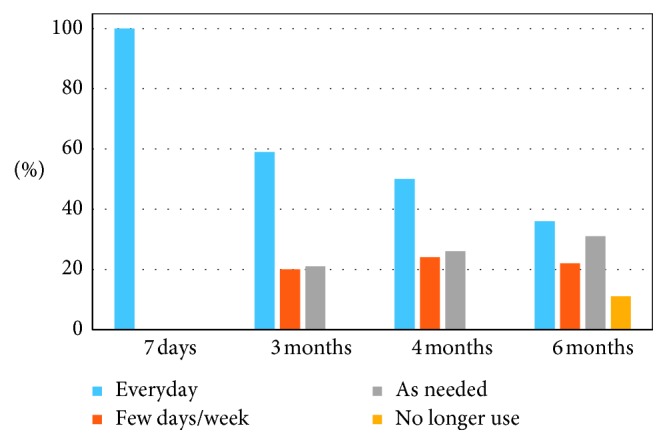
Device usage patterns among study sample cohorts over the 6-month period. Pain relief was maintained over the 6-month period despite decreasing device use. The number of subjects using the device daily decreased from 100% in the first 7-days, to only 36% at 6-months. Additionally, 11% of users no longer needed to use the device after 6 months.

Usage of the medical device monotonically decreased over time, from 100% using the device every day during the first 7-day period, to only 36% using the device every day after 6 months. Forty-one percent (41%) reported using the device only as needed or stopped using it completely by the end of the study (Figure 3).

In terms of overall improvements for the study sample, 62% reported a “*great deal*” or “*definite improvement*” in their QoL. For sleep, 60% reported a “*great deal*” or a “*fair amount*” of improvement, while for physical improvement, 53% reported a “*lot more*” or a “*fair amount*” of improvement. For medication, 52% reported a “*lot*” or a “*fair amount*” of reduction. These four percentages of people reporting meaningful improvements were compatible with the 57% of the sample who reported having mild levels of final pain (defined as having pain levels of 3 VAS points or less). They are also in line with the 73% who reported at least a 50% reduction in pain and the 52% who reported at least a 60% reduction in pain by the end of six months of treatment.

### 3.6. Likeliness of Long-Term Pain Relief

Linear regression analyses were used to identify the observable variables that could best predict which subjects were most likely to receive long-term pain relief. Pain relief (dependent variable) was defined in terms of seven different measures: the first three were in terms of VAS scores, i.e., final pain level, change in pain, and percent improvement, while the remaining four were based on the two function measures, sleep and physical activity, the one being change in QoL measure and the other being measure of change in medication use. As a result, there was a total of 7 independent regression analyses conducted, each tapping the underlying construct of pain relief. In all cases, the same 13 independent variables were used. Variables are categorized in terms of five subsets: demographics, etiology and location of pain, baseline pain intensity, baseline treatments, and the 7-day pain level (Table 7).

This selection of variables was done for three reasons. First, all these variables are available after the subject used the PSWT device for the initial 7-day treatment. Second, by including a broad set of predictors, it is possible to control for the diverse set of etiologies, baseline conditions, and use of other therapies in determining long-term pain relief. Third, by including all the variables in each analysis, it is possible to better assess if any discovered association is possibly spurious or consistent across the multiple dimensions of pain relief.

The regression coefficients and the statistical significance of factors that reached at least 0.05 level of significance are shown in Table 7. Consistent with Table 5 results, the VAS score following the 7-day treatment is a statistically significant predictor for all seven pain relief measures. Higher 7-day VAS scores are indicative of lower long-term treatment effectiveness, regardless of which of the seven pain relief measures were used. No other observable variable following the 7-day treatment had a strong predictive value across all the multiple pain relief measures. Higher baseline VAS levels were associated with a greater reduction in pain (VAS change both in absolute and percent), greater improvements in sleep quality, and overall QoL by the end of the study, all else equal. The longer a subject suffered with a pain condition (duration), the higher their VAS scores tended to be after the 6-month intervention. Longer pain durations were also found to have a negative impact on changes in medication use, as does the use of other different treatment therapies. In contrast, reduction in medication use tends to be greater for subjects who initially (at baseline) used more OTC medications and/or were less likely to use other therapies prior to using the PSWT device. The only demographic variable to reach significance was the age of the subject which was negatively correlated with the person's change in QoL. Other than fibromyalgia, which is negatively associated with change in sleep quality, none of the seven pain relief measures were found to be related to the location or etiology of the pain.

## 4. Discussion

There is inadequate evidence to ascertain whether over-the-counter electroceutical technologies, such as TENS, are effective in relieving chronic pain [28]. Pulsed shortwave therapy (PSWT) is an OTC electroceutical technology that uses electromagnetic fields (EMFs) to achieve nonsensory neuromodulation without skin contact, thus allowing continuous and recurring use. In this study, we investigated the durability of PSWT treatment in relieving chronic pain.

The PSWT device that was used in the present study was previously evaluated for treatment effectiveness in two large registry studies [22, 23]. In both studies, it was found that about 2/3^rd^ of the users obtained clinically significant reductions in pain (VAS reduction ≥ 2). In addition, it was also shown that this pain reduction was obtained for multiple etiologies and anatomical locations. However, these registry studies did not evaluate whether the pain reduction reported by these subjects was durable, and if so, whether it was possible to predict which subset of subjects were most likely to experience treatment durability. Evaluating treatment durability is important for any medical intervention, since a vast body of clinical research indicates that many pharmacological treatments show a decline in effectiveness over time [26, 27]. This lack of efficacy from a wide range of existing treatments was evident among subjects in the two discussed registry studies as well as the present study, as witnessed by the fact that subjects reported high baseline pain levels (VAS ≥ 7) despite actively using one or more analgesic therapies [22, 23]. The present prospective study examines the durability of treatment effectiveness for a PSWT neuromodulation device in a cohort of 240 subjects who had indicated clinically significant pain reduction after using the PSWT device for 7 days.

The 240-subject sample recruited for this study did not present with any significant differences in age, duration of pain, and baseline pain level from the sampled population of 1841 UK/Ireland chronic pain sufferers. Both groups had a high incidence of women participants, with 66% in the sampled population and 70% in the study sample classifying themselves as women. The slightly larger percentage in the study sample was due to the fact that 66% of all the women in the total population indicated a clinically significant reduction in pain after 7 days of treatment compared to only 55% for men and thus were more likely to be asked to participate in the study. This finding of women being more likely to report clinically significant reduction in pain is in line with the prior literature that indicates differences in responses to pain between men and women [29, 30] and merits further exploration for why such differences might occur. Both the sampled population and study samples reported a wide range of etiologies and pain locations although arthritis and fibromyalgia were the most common pain etiologies and back was the most common location. The study sample of 240 subjects reported a higher incidence of fibromyalgia (20%), when compared to 10% for the population of 1841 chronic pain sufferers. We cannot ascertain at this time why the study group had a higher incidence of fibromyalgia participants. There were only minor differences in average age and pain duration between subjects reporting a clinically significant reduction in pain after the 7-day treatment and those that did not. The implication is that these variables are not good predictors of who are most likely to report 7-day pain relief from the focal medical device.

The range of pain etiologies and other demographic information measured within the study sample allowed investigation of factors that could be highly associated with both initial pain relief and the durability of this relief. The initial pain relief attributed to treatment from the focal medical device is not highly associated with any etiology of pain or demographic factor, other than gender (Table 2). The regression analyses revealed that the only statistically significant factor in predicting the seven six-month pain relief measures was an individual's 7-day pain score. This is noteworthy, since it highlights that if this 7-day treatment is successful, the individual is highly likely to continue to get long-term pain relief, not only in terms of reduced pain levels but also in terms of increased function and decreased medication use. Pain duration at baseline was also a good predictor for assessing final pain levels, level of pain reduction (percent), and change in medication use—the longer the pain duration, the lower the reported pain relief. Thus, it appears that the longer a subject was suffering from pain, the less relief the person was able to obtain. Interestingly, no gender effects were noted, and the only pain etiology found to predict six-month pain relief was fibromyalgia, which negatively impacted improvements in sleep quality. Thus, neither etiology nor location of pain appears to have any major influence in determining who will get pain relief (either short term or long term), with gender only affecting the probability of getting initial pain relief (women more so, than men). Conditional on getting this relief, no gender differences were found in terms of long-term relief.

The consistency of the treatment effectiveness over the six-month period is reflected in the trend of the mean VAS reduction, which after the 7-day treatment was 5.3 points (65% reduction) and 4.9 points (60% reduction) after six months. In terms of pain reduction, the majority (73%) of the study sample reported at least 50% VAS reduction compared to baseline at the end of the 6-month period and more than half the sample (52%) reported pain reduction of at least 60%. When categorized by VAS scores at the end of the 6-month intervention, 57% of the sample previously suffering with severe chronic pain reported they were only in mild pain, while 29% reported moderate pain levels. These results were found to be independent of the pain etiology and pain location.

When tracking the consistency of an individual's pain relief over time, we found that 72% of subjects who report their pain after the 7-day treatment as mild will continue to experience this level of pain relief even at the end of the 6-month period. The same pattern was observed for subjects reporting moderate pain after 7-day treatment, with 83% of these subjects reporting mild-moderate pain after 6 months. Interestingly, 91% of the few subjects in our sample who reported severe pain even after the 7-day treatment were no longer in severe pain by the end of 6 months, with 36% experiencing only mild pain and 55% experiencing moderate pain. This indicates that a majority of individuals who reported still being in severe pain even after reducing their baseline pain by 2 or 3 VAS points after the initial 7-day treatment still benefited from longer-term PSWT use. However, this latter group was composed of only 14 subjects, and thus, we caution the reader not to generalize from these findings. Further study is needed to determine if longer use of the medical device is needed for some subjects who did not get initial significant pain relief to get subsequent long-term functional improvements. With this noted, the overarching finding is that the long-term benefits of PSWT for chronic pain patients in terms of pain reduction can be quickly assessed after a relatively short trial period of 7 days. Once assessed, patients who report clinically significant pain relief with the 7-day initial treatment have a high likelihood of maintaining the pain relief for at least six months.

Unsurprisingly, pain reduction was accompanied with functional improvements in sleep quality, physical activity, patient global impressions of life changes, and a reduction in medication use—all of which are strong indicators of QoL improvements. This decrease in analgesic medication use over the 6-month period is a significant outcome, since long-term use of many analgesics results in adverse side effects that can impact patient quality of life [23, 31]. These include the highly significant and often multiple adverse side effects from opiate-based analgesics that are correlated with higher doses and long-term use [32].

The PSWT device used in this study was a low-power RF electroceutical that provided therapeutic benefits through peripheral neuromodulation [19, 20]. This is in line with Brook et al. [21] who demonstrated that neuromodulation can occur when low-power RF transmitters, such as the one used in the PSWT study device, are operated adjacent to biological tissue and at maximum output [18]. It is also compatible with clinical evidence showing that PSWT stimulation increases proximal and distal pain tolerance thresholds in subjects with knee osteoarthritis [24] and is consistent with the premise that mitigation of nerve hypersensitivity plays a critical role in treating chronic, intractable pain. Additionally, PSWT treatment has also been shown to reduce pain for patients presenting with various levels of nerve hypersensitivity (central sensitization), as measured by a standardized, central sensitization inventory assessment (CSI) [33–35], an evaluation tool developed to determine the extent of CS in chronic pain patients [34, 35].

The durability results reported from the current study indicate that subjects do not appear to habituate to continuous/recurring PSWT stimulation. This may be a reflection of the mechanism of action associated with PSWT, i.e., the *stochastic* (i.e., nondeterministic) nature of the stimulation. Moreover, subjects continued to maintain lower pain levels over the study period despite gradually reducing the duration of device use. This may be due to an increase in pain tolerance thresholds, indicating a possible mitigation of underlying nerve hypersensitivity associated with the chronic pain etiology. Whether PSWT treatment can mitigate central sensitization itself needs to be further evaluated in future randomized, double-blinded, placebo-controlled studies by utilizing CSI as an outcome measure.

### 4.1. Possible Limitations

This study has several limitations. First, the study utilized self-reported data and some of the measures required the subject to recall levels of pain, sleep, etc., prior to using the PSWT device. Consequently, these measures are subject to recall bias. However, it is the authors' belief that since chronic pain is salient to the respondents, recalling pain levels is not a cognitively difficult task. Perhaps of more concern is the reliability of responses, especially for pain. However, the average responses for subjects were consistent with findings from the large-scale registry studies discussed earlier. Likewise, many of the measures used in the study recorded changes over time, e.g., the difference in baseline and final pain (6 month) levels. By utilizing differences, any individual level bias associated with the person over/under reporting pain levels was removed. Other change measures were taken relative to the person's long-term baseline. These function and medication measures trended in the same direction as the person's pain reduction, thereby increasing confidence that the study captured true therapeutic responses to the PSWT treatment.

Another possible limitation is the lack of a control arm. Placebo effects associated with analgesics have been reported to range from 19% to 30% [36] and result in an average reduction of about 1.5 VAS points [37]. Furthermore, prior placebo-controlled studies using the same PWST device have shown only modest placebo responses [21, 24]. The observed reduction in pain over the six-month study was 59% (4.9 VAS points), which is far greater than the reduction associated with analgesic placebo effects reported in the literature. Moreover, the authors are unaware of any clinical research indicating that placebo effects associated with an active analgesic intervention can persist for 6 months, in the majority of subjects under study. While the decrease in pain level and an increase in functionality/QoL over the 6-month period could be attributed to causes other than the medical device, it is to be noted that these subjects had experienced persistent pain for several years and tried multiple interventions—without obtaining substantial and/or sustained pain relief. Thus, there is little reason to expect that this pain relief fortuitously occurred in the study period.

## 5. Conclusion

Electroceuticals offer immense potential as a nonpharmacological intervention for chronic pain management. Current over-the-counter electroceuticals, such as TENS devices, rely on skin contact to achieve neuromodulation. However, continuous/recurring use of TENS is limited due to the potential for skin damage, need for short use duration (typically, less than 30 mins, twice a day), and unpleasant sensations (shocks, tingling). PSWT electroceuticals, on the other hand, use electromagnetic fields (EMF), which easily pass through skin/bandaging, can be incorporated into wraps/braces and are well tolerated by patients owing to a lack of any sensation during use. This prospective study involved a 6-month assessment of 240 chronic pain subjects, who at the time of enrollment had obtained pain relief after 7 days of treatment with a commercially available PSWT electroceutical device. The results indicate that pain relief was sustained for 6 months in over 85% of subjects. In addition, subjects reported a substantial improvement in functionality through measures such as physical activity, sleep quality, and overall quality of life. They also decreased consumption of pain medication, including prescription and opioid-based pain medications.

A major objective of any electroceutical is to serve as an effective adjunct for multimodal pain management. PSWT was found to be consistently effective in providing pain relief for varying pain etiologies and in multiple anatomical locations. Given the lack of adverse effects and ability of patients to tolerate long-term PSWT use, it is the authors' conclusion that PSWT is an effective, over-the-counter electroceutical therapy for a substantial portion of the chronic pain population.

## Figures and Tables

**Table 1:** Available responses and coding for assessment queries.

Assessment queries	Response options
How often do you use ActiPatch?	(i) Every day = 1 (ii) A few times a week = 0.5 (iii) only when needed = 0.3
Pain level (11-point VAS scale)	(i) No pain = 0,………….., worst pain = 10 (ii) *Mild pain is defined as scores of 0-3, moderate as pain scores of 4–6, and severe as pain scores of 7–10*
Changes in sleep and physical activity relative to prior use of ActiPatch	(i) No change = 0 (ii) Increased a little = 1 (iii) Increased a fair amount = 2 (iv) Increased a lot = 3
Changes in medication use relative to prior use of ActiPatch	(i) No change = 0 (ii) Decreased a little = 1 (iii) Decreased a fair amount = 2 (iv) Decreased a lot = 3 (v) Stopped using medications = 4
Patient global impressions of change with PSWT treatment	(i) No change or got worse = 0 (ii) Almost the same, but hardly any change at all = 1 (iii) A little better,but no noticeable difference = 2 (iv) Somewhat better, but the change has not made any real difference-3 (v) Moderately better, a slight but noticeable change = 4 (vi) Better, a definite improvement that has made a real and worthwhile difference = 5 (vii) A great deal of better and considerable improvement that has made all the differences in the world = 6

**Table 2:** Baseline demographics/etiologies between total sample and study sample.

	Sample population (*n* = 1841)	Sample with ≥2 VAS reduction at 7 days (*n* = 1143)	Sample with <2 VAS reduction at 7 days (*n* = 698)	Sample with ≥2 VAS reduction at 7 days + “definitely purchase” (*n* = 682)	Sample with ≥2 VAS reduction at 7-days + “not definitely purchase” (*n* = 461)	Study sample (*n* = 240)
*Demographics*						
Age (years)	55.6	54.4	54.3	56.4	56.3	57.9
Duration of pain (years)	6.4	6.5	6.2	6.4	6.6	6.5
Women	66%	70%	61%	71%	69%	70%
Pain > than 6 months	89%	89%	88%	90%	88%	91%
Baseline VAS	8.02	8.17	7.77	8.26	8.02	8.23
7-day treatment VAS	5.03	3.38	7.72	2.97	3.97	2.82
% pain reduction	33%	59%	0%	64%	51%	66%
VAS ≥2 reduction	62%	100%	0%	100%	100%	100%
VAS ≥3 reduction	54%	87%	0%	94%	77%	97%
% “definitely purchase” intent	38%	60%	3%	100%	0%	100%
*Pain etiology*						
Osteoarthritis	30%	31%	28%	33%	26%	25%
Rheumatoid arthritis	15%	15%	14%	15%	15%	7%
Fibromyalgia	10%	10%	9%	10%	11%	20%
Sports injury	8%	8%	7%	8%	8%	12%
Neuropathy	5%	5%	5%	5%	5%	8%
Surgery	6%	6%	5%	6%	6%	6%
Tendinitis	3%	3%	3%	3%	2%	4%
Other	23%	22%	29%	20%	27%	18%

Subjects often reported pain in multiple areas of the body, but this designation was predominantly for the back (49%), followed by knee (27%), shoulder (17%), hip (16%), neck (8%), and others (11%). The rank ordering of the locations where the PSWT medical device was applied mirrored the rank order of reported locations of pain.

**Table 3:** Analgesic medications used by the study sample.

Analgesics	Fraction of users (%)
NSAIDS (e.g., ibuprofen)	43
Paracetamol	61
Weak opioids (e.g., codeine)	25
Strong opioids (e.g., hydrocodone)	11
Tramadol or equivalent	8
Pregabalin (e.g., Lyrica)	8
Amitriptyline	17
Topical opioid (e.g., morphine)	8
Topical NSAIDS (e.g., Voltarol)	21
Gabapentin	2
Other	4
No analgesics	5

Note: medication use data were available for only 172 out of the 240 subjects.

**Table 4:** VAS scores of the study sample categorized by mild, moderate, and severe levels at baseline and at the four assessment periods.

Pain score (VAS)	Fraction of study sample					
	Baseline (*N* = 240)	7 days (*N* = 240)	3 months (*N* = 222)	4 months (*N* = 208)	6 months (*N* = 209)	Last reported (*N* = 240)
0–3 (mild pain) (%)	0	70	67	61	58	57
4–6 (moderate pain) (%)	9	27	30	35%	36	37
7–10 (severe pain) (%)	91	3	3	4	6	6
Average sample VAS	8.23	2.86	2.96	3.13′	3.25	3.31

The last column is data from the last-observed value of all subjects.

**Table 5:** Transition matrix for VAS score.

VAS score after initial PSWT treatment (7 days)	VAS score at end of study (6 months)		
	0–3 (mild pain) (%)	4–6 (moderate pain) (%)	7–10 (severe pain) (%)
0–3 (mild pain)	72	28	0
4–6 (moderate pain)	31	52	17
7–10 (severe pain)	36	55	9

A majority of subjects who are experiencing only mild pain after the initial 7-day PSWT treatment continue to maintain relief over 6 months. The same is true for individuals with mild pain although there is more of a tendency to see a reduction in pain versus an increase. The majority of subjects still in severe pain after 7 days of PSWT treatment reduced their pain over 6 months.

**Table 6:** Average outcome measures for the different levels of final pain.

Last reported VAS	% study sample (*N* = 240)	7-day VAS (after 7-day treatment)	Δ VAS (baseline: last reported)	Δ QoL (baseline: last reported)	Δ sleep (baseline: last reported)	Δ medications (baseline: last reported)	Δ physical activity (baseline: last reported)	% stopped medication use	% stopped nondrug pain therapies
0–1	18%	1.43	7.41	+5.48	+2.22	+3.02	+2.18	34	23
2–3	39%	2.71	5.54	+5.07	+1.99	+2.5	+1.86	16.5	17
4–5	28%	3.59	3.91	+4.6	+1.76	+2.02	+1.4	13.5	14
6–7	12%	4.31	2.58	+3.95	+1.23	+0.57	+0.98	0	13
8–10	3%	5.00	−0.33	+3.50	+1.00	+0.50	+0.83	0	0

Numerical values for the change measures can be found in Table 1.

**Table 7:** Variables used in the regression analyses to determine if the variable is useful in explaining a particular outcome measure and the coefficients and significance level of those which reached the 0.05 significance level.

Independent variable	Final pain score	Change in pain	% improvement	Change in sleep	Change in physical activity	Change in QoL	Change in meds
*Demographics*							
Gender (women)							
Age						−0.013 (0.1)	
*Etiology/location*							
Arthritis							
Fibromyalgia				−0.364 (0.03)			
Back							
Knee							
Other location							
*Pain intensity*							
Baseline		1.10 (00)	0.058 (00)			0.112 (0.04)	0.153 (0.02)
Duration	0.045 (00)	−0.044 (00)	−0.005 (02)				−0.038 (0.00)
*Current treatment*							
OTC meds							0.356 (00)
Use of opiate meds							
Other therapies							−0.186 (0.01)
7-day pain level	0.613 (00)	−0.633 (00)	−0.074 (00)	−0.079 (0.04)	−0.080 (0.04)	−1.87 (00)	−0.264 (00)
*R* ^2^	0.36	0.48	0.32	0.10	0.08	0.18	0.27

## Data Availability

The data are the property of BioElectronics Corp but can be viewed on request.
